# Gene expression profiling of fibroblasts in a family with *LMNA*-related cardiomyopathy reveals molecular pathways implicated in disease pathogenesis

**DOI:** 10.1186/s12881-020-01088-w

**Published:** 2020-07-22

**Authors:** Halida P. Widyastuti, Trina M. Norden-Krichmar, Anna Grosberg, Michael V. Zaragoza

**Affiliations:** 1 UCI Cardiogenomics Program, Department of Pediatrics, Division of Genetics & Genomics and Department of Biological Chemistry, University of California, Irvine, School of Medicine, 2042 Hewitt Hall, Irvine, CA 92697-3940 USA; 2 Department of Epidemiology, University of California, Irvine, School of Medicine, 3062 Anteater Instruction and Research Building, Irvine, CA 92697-7550 USA; 3 Department of Biomedical Engineering and The Edwards Lifesciences Center for Advanced Cardiovascular Technology, University of California, Irvine, Irvine, California USA

**Keywords:** *LMNA* gene, Lamin, Laminopathies, Cardiomyopathy, RNA sequencing, *IGFBP5* gene, Fibroblasts, Gene expression, Signaling pathway

## Abstract

**Background:**

Intermediate filament proteins that construct the nuclear lamina of a cell include the Lamin A/C proteins encoded by the *LMNA* gene, and are implicated in fundamental processes such as nuclear structure, gene expression, and signal transduction. *LMNA* mutations predominantly affect mesoderm-derived cell lineages in diseases collectively termed as laminopathies that include dilated cardiomyopathy with conduction defects, different forms of muscular dystrophies, and premature aging syndromes as Hutchinson-Gilford Progeria Syndrome. At present, our understanding of the molecular mechanisms regulating tissue-specific manifestations of laminopathies are still limited.

**Methods:**

To gain deeper insight into the molecular mechanism of a novel *LMNA* splice-site mutation (c.357-2A > G) in an affected family with cardiac disease, we conducted deep RNA sequencing and pathway analysis for nine fibroblast samples obtained from three patients with cardiomyopathy, three unaffected family members, and three unrelated, unaffected individuals. We validated our findings by quantitative PCR and protein studies.

**Results:**

We identified eight significantly differentially expressed genes between the mutant and non-mutant fibroblasts, that included downregulated insulin growth factor binding factor protein 5 (*IGFBP5*) in patient samples. Pathway analysis showed involvement of the ERK/MAPK signaling pathway consistent with previous studies. We found no significant differences in gene expression for Lamin A/C and B-type lamins between the groups. In mutant fibroblasts, RNA-seq confirmed that only the *LMNA* wild type allele predominately was expressed, and Western Blot showed normal Lamin A/C protein levels.

**Conclusions:**

*IGFBP5* may contribute in maintaining signaling pathway homeostasis, which may lead to the absence of notable molecular and structural abnormalities in unaffected tissues such as fibroblasts. Compensatory mechanisms from other nuclear membrane proteins were not found. Our results also demonstrate that only one copy of the wild type allele is sufficient for normal levels of Lamin A/C protein to maintain physiological function in an unaffected cell type. This suggests that affected cell types such as cardiac tissues may be more sensitive to haploinsufficiency of Lamin A/C. These results provide insight into the molecular mechanism of disease with a possible explanation for the tissue specificity of *LMNA*-related dilated cardiomyopathy.

## Background

The Lamin A/C (*LMNA*) gene encodes for Lamin A and Lamin C proteins that, along with Lamin B1 and Lamin B2, form an intricate intermediate filament protein meshwork termed the nuclear lamina (NL) and play important roles in maintaining nuclear structure and stability and in fundamental nuclear functions [1–3]. Lamins A and C proteins are products of alternative splicing of the *LMNA* gene. Lamin A is produced from all twelve exons of the gene, while Lamin C is the product of only ten exons. In vivo, Lamin A is produced as prelamin A and undergoes extensive post-translational processing of the C-terminus to become mature Lamin A protein while Lamin C is produced as mature protein [4]. Aside from conveying structural integrity to the nucleus [5], nuclear lamina proteins associate with heterochromatin [6], modulate gene expression by sequestering transcription factors to the nuclear periphery [7], regulate cell cycle progression [8], and regulate molecular signaling such as ERK/MAPK and Wnt Beta-Catenin pathways [9–11]. The diverse roles of the nuclear lamina proteins underscore their importance in maintaining proper cellular function in an organism.

Despite having common functional roles, the expression patterns of Lamin A/C, Lamin B1, and Lamin B2 are different [12]. Lamin A/C expression is absent in human and mouse embryonic stem cells but increases once these cells are induced to differentiate [13]. In contrast, Lamin B1 and Lamin B2 expression are ubiquitous throughout development. Lamin B1 is highly expressed in the embryo and its expression persists throughout development [14]. Similarly, Lamin B2 is expressed early during development, and its expression remains ubiquitous during development [15]. Additionally, Lamin A/C expression levels vary among differentiated cell types. They are more highly expressed in multinucleated cells, such as cardiomyocytes, compared to mononucleated cells, such as fibroblasts [12, 16, 17].

Although Lamin A/C is expressed in most differentiated cell types, *LMNA* mutations predominantly affect mesoderm-derived cell lineages in diseases collectively termed as laminopathies. Laminopathies range from dilated cardiomyopathy (DCM) with conduction defects [18–20] and different forms of muscular dystrophies, such as Emery-Dreyfus Muscular Dystrophy (EDMD) [21–23] and Limb Girdle Muscular Dystrophy type 1B (LGMD1B) [24], to severe disease characterized by premature aging such as Hutchinson-Gilford Progeria Syndrome [25, 26]. *LMNA*-related DCM with conduction defects is one of the most common forms of inherited dilated cardiomyopathy, second only to DCM associated with mutations in sarcomere protein genes [27, 28], with an estimated 5 to 10% of cases associated with a heterozygous *LMNA* mutation [29–31]. Current hypotheses for how *LMNA* mutations give rise to diseases limited to cardiac tissues include the mechanical defect and the gene expression hypotheses [32]. The mechanical defect hypothesis proposes that *LMNA* mutation compromises the structural integrity of a cell, subsequently causing the cells to be more prone to necrosis leading to diseases [33]. The gene expression hypothesis proposes that *LMNA* mutation impairs the NL structure which leads to aberrant epigenetic modification, abnormalities in signaling transduction and ultimately affecting proper gene expression [32, 34]. As of now, it remains unclear which of these mechanisms is the main cause of *LMNA*-related cardiomyopathy.

We recently identified a novel splice-site mutation in the Lamin A/C gene, *LMNA* c.357-2A > G (p.N120Lfs*5), in a multigenerational family with DCM, heart failure, and sudden death [35]. In this study, we performed RNA sequencing (RNA-seq) to test the hypothesis that the splice-site mutation in the *LMNA* gene is associated with altered expression of genes that played a significant role in the nuclear lamina structure and function. By deep RNA-seq of patient and control fibroblasts, we evaluated expression of Lamins A and C, tested for aberrant splicing of *LMNA* transcripts, and altered ratio of *LMNA* transcript variants. We also sought to determine any compensatory mechanism by Lamin B1 and/or Lamin B2 in response to the mutation.

We further expanded the analyses to examine the most significantly differentially expressed genes across the genome, and determine potential pathways affected by the *LMNA* mutation or other genes and pathways influencing the observed phenotype. Surprisingly, from the RNA-seq data we did not find any significant differential expression for the Lamin isoforms between the sample groups. However, we found plausible genes and pathways that may contribute to the cardiomyopathy phenotype that we observe in this family. Our study highlighted tissue specificity as a major feature of the novel heterozygous *LMNA* splice-site mutation as evidenced by the lack of changes in nuclear lamina gene expression and absence of aberrant splicing events in patient fibroblasts. The study conducted here laid the foundation for future disease modeling studies in cardiomyocytes to elucidate on the molecular mechanism of the novel *LMNA* splice-site mutation.

## Methods

### Fibroblast collection

Nine primary fibroblast cell lines were cultured from skin biopsies (Table 1) as previously described [35]. These included cells from six family members: three affected individuals (Patient 1–3) heterozygous for the *LMNA* splice-site mutation and three unaffected individuals (Control 1–3) who do not have the *LMNA* splice-site mutation. To serve as Unrelated Controls (U), fibroblast cells from three healthy individuals were obtained from biorepositories (U1: Lonza CC-2511; lot#: 0000352805; U2: Lonza CC-2511; lot#: 0000293971; U3: Coriell Institute ND31845). To confirm the *LMNA* genotype, genomic DNA (gDNA) was extracted from Patient, Control, and Unrelated Control fibroblasts and evaluated by Sanger sequencing of the 12 *LMNA* exons as previously described [35]. The *LMNA* genotype for each sample was also confirmed by examining the RNA-seq data.

**Table 1:** Fibroblast cell samples that were RNA sequenced (*N* = 9)

Identification	Lamin A/C Genotype*	Age (years) at Cardiomyopathy Diagnosis	Age (years) at Skin Biopsy
Patient 1 (P1)	+/−	36	38
Patient 2 (P2)	+/−	58	62
Patient 3 (P3)	+/−	61	70
Control 1 (C1)	+/+	**–**	49
Control 2 (C2)	+/+	**–**	69
Control 3 (C3)	+/+	**–**	68
Unrelated Control 1 (U1)	+/+	**–**	40
Unrelated Control 2 (U2)	+/+	**–**	51
Unrelated Control 3 (U3)	+/+	**–**	73

*+/+ homozygous normal allele; +/− heterozygous *LMNA* splice-site mutation

### RNA-seq

Total RNA from Patient, Control, and Unrelated Control fibroblasts (at passage 7) was isolated and quantified as described [35]. RNA-seq studies were conducted on total RNA (3 to 7 μg) at DNA Link USA, Inc. (San Diego, CA) using poly-A RNA enrichment and library preparation. RNA libraries were sequenced as 75 bp paired-end runs with at least 100 million reads per sample on an Illumina NextSeq 500 platform (Additional File 1: Table S1). The raw RNA-seq data (fastq) was stored and transferred using the BaseSpace Sequence Hub (Illumina, San Diego, CA).

### Bioinformatics analysis

#### Quality control, alignment, and differential expression (DE)

RNA-seq data was first examined for quality using FastQC software [36]. Reads were filtered out due to low quality. Low quality bases at the 3′ and/or 5′ ends of the reads were trimmed. The reads that pass the quality filtering were aligned to the human reference genome (GRCh37/hg19) with TopHat2 alignment software [37]. The average overall read mapping rate was 90.1% and average concordant pair alignment rate was 85.5% (Additional File 1: Table S1). DE analysis was performed with the Cufflinks software [38, 39], using upper quartile normalization between the data files. Normalized DE genes between the groups were filtered for absolute fold change ≥1.5, Fragments Per Kilobase of transcript per Million mapped reads (FPKM) ≥ 1, and that were significant at false discovery rate (FDR)-adjusted *p*-value ≤0.05. DE of isoforms was provided by cufflinks/cuffdiff2 software to distinguish between the expression of Lamin isoforms. From this list of significantly DE genes, we further filtered the list to include only DE genes that were expressed similarly between the Unrelated and Patient groups and between the Control and Patient groups. Our rationale to filter the genes using these parameters was to find genes that potentially were affected by the *LMNA* splice-site mutation. We reasoned that DE genes that were shared between Control and Unrelated groups were due to intrinsic gene expression differences and not due to the mutation. By looking only at genes that were at the intersection of Unrelated vs. Patient and Control vs. Patient groups, we narrowed down the candidate genes to those that potentially were affected by the mutation.

#### Visualization and pathway analysis

Heatmaps of the gene expression in FPKM values of the most highly DE genes were constructed with cummeRbund [40]. Heatmaps were clustered by rows, where each row contained the gene expression of a particular gene. Lamin isoforms were visualized in heatmaps along with any other genes of interest. To detect allelic expression of Lamin A/C transcripts, we used the Integrative Genomics Viewer (IGV) software [41] to visualize the distribution of alleles in mapped reads at rs538089 in Exon 5 and rs4641 in Exon 10 for samples found to be heterozygous by Sanger sequencing of gDNA [35]. Finally, Ingenuity Pathway Analysis (IPA) software (Qiagen, Hilden, Germany) was used to determine the top canonical pathways and visualize the significant networks.

### qPCR validation of RNA-seq results

Quantitative PCR (qPCR) assays were performed to validate RNA-seq results for Lamin A, Lamin C, Lamin B1, Lamin B2, and IGFBP5. cDNA was synthesized using QuantiTect Reverse Transcription Kit (Qiagen) from Unrelated, Control and Patient RNA samples. 100 ng/μl cDNA was used as the reaction template with 10 μM of pre-designed Kicqstart forward and reverse primer pairs (Sigma Aldrich, St. Louis, MO) specific to Lamin A and Lamin C, 10 μM of independently designed primer pairs for Lamin B1, Lamin B2, and IGFBP5 (Integrated DNA Technologies, San Diego, CA), and SYBR green dye along with the necessary reaction components (KAPA Biosystems, Wilmington, MA). The reaction was run in three technical replicates for each sample. Comparative Ct (ΔCt) method was used to determine relative quantitative gene expression (QGE) [42, 43]. Beta Actin (*ACTB*) was used as the housekeeping gene. Statistical analysis was performed using One-Way ANOVA followed by Tukey post hoc test to determine statistical significance between groups. *P* values less than 0.05 were considered statistically significant. Primer sequences used for validation are provided in Additional File 1: Table S2.

### Lamin A/C protein level validation

Patient, Control and Unrelated Control fibroblasts were grown to confluency, harvested using TrypLE Select 1X (Thermo Fisher Scientific, Waltham, MA), and lysed using cold RIPA buffer supplemented with protease inhibitors cocktail (Sigma-Aldrich, Saint Louis, MO). Protein concentration was quantified using Pierce™ BCA Protein Assay Kit (Thermo Fisher Scientific). 50 μg of total protein lysate along with protein ladder were run on a Bolt™ 4–12% Bis-Tris Plus Gels (Thermo Fisher Scientific) under denaturing conditions followed by wet transfer. Target protein was detected using a primary antibody for Lamin A/C (sc-376248, Santa Cruz Biotechnology, Dallas, TX), at 4 °C overnight followed by incubation with secondary antibody conjugated to HRP (1:5000, Abcam, Cambridge, UK) for one hour at room temperature. Beta Actin was used as loading control. Protein visualization was conducted using iBright FL1000 Imaging System and relative quantification of protein bands was performed using ImageJ [44].

## Results

### Expression variability in samples within and between groups

**Fig. 1:**
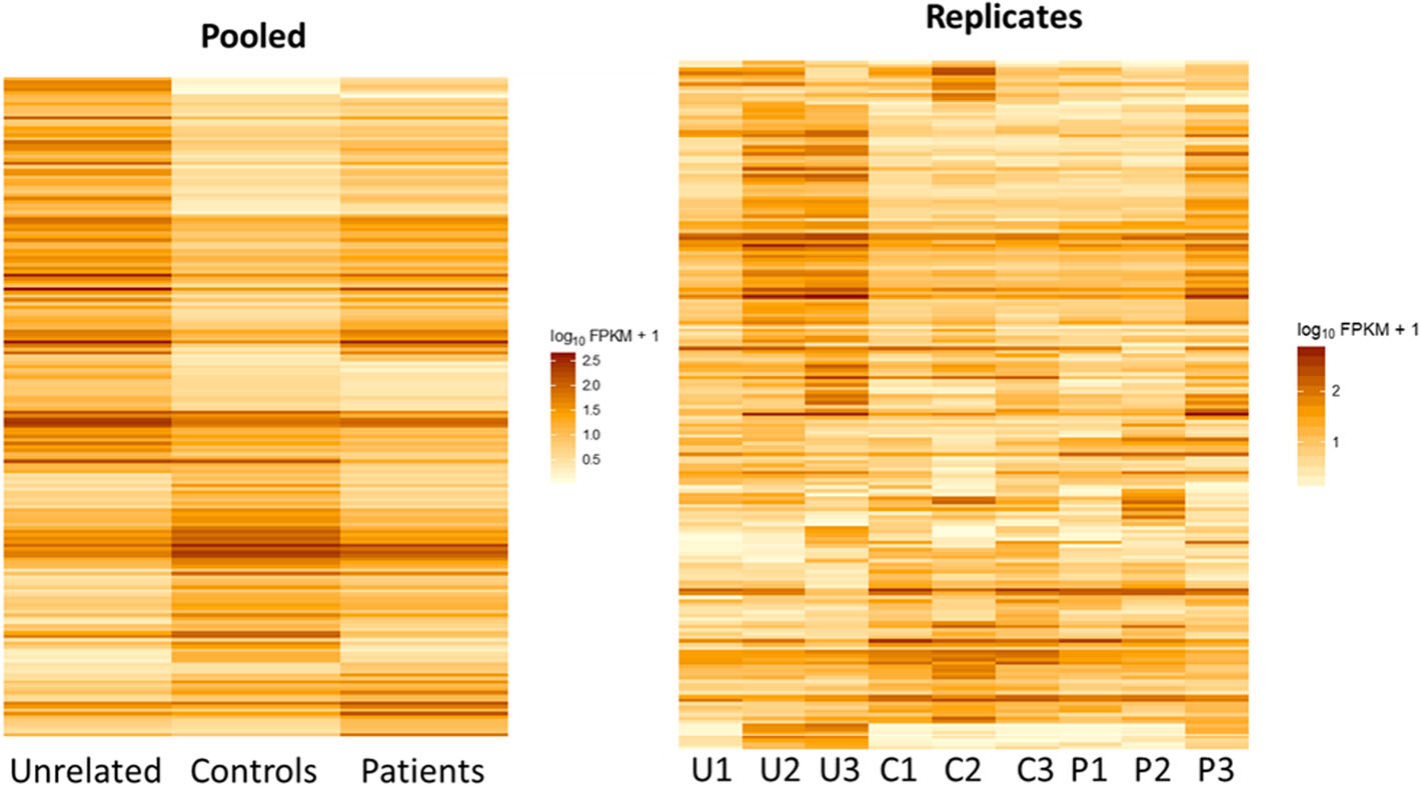
Gene expression profiling of fibroblast samples by RNA-seq. Differential gene expression analysis was performed using Tophat and Cufflinks software and visualized using cummeRbund. Heatmaps of gene expression profiles are clustered by rows (genes). Heatmaps are shown as pooled samples (left panel) and replicates (right panel)

In this study, we used whole transcriptome DE analysis to investigate the genes and pathways related to *LMNA*-related cardiomyopathy. Differences between the gene expression in the pooled sample groups (Unrelated, Control, Patient) were observed by plotting the top DE genes (Fig. 1, left panel). By plotting gene expression by replicate, we observed variation within each sample group (Fig. 1, right panel) and found that Patient and Control groups were more similar to each other, compared to samples in the Unrelated Control group. The heatmaps show the heterogeneity between the samples, where the overall trend was that gene expression pattern of the Patient group was more similar to that of the Control group.

### Unaltered NL-associated gene and protein expression in patient fibroblasts

**Fig. 2:**
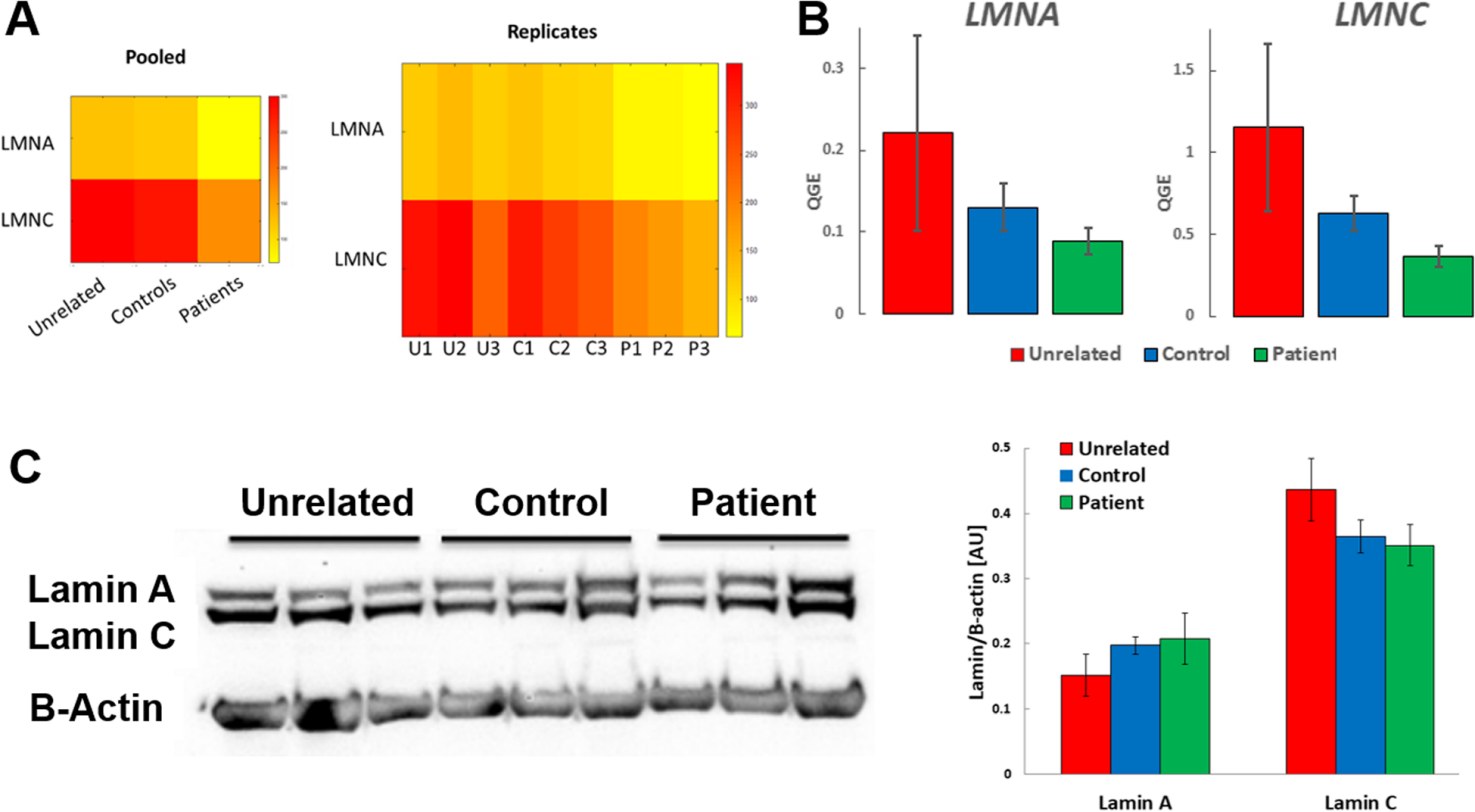
Nuclear lamina-associated gene expression profiling across all samples. **a** Visualization of Lamin A and Lamin C expression across all samples from RNA-seq data. Lamin A and Lamin C expression obtained from RNA-seq experiments were visualized using heatmaps. Gene expression is shown pooled (left panel) and as replicates (right panel). In general, Lamin A and Lamin C expression are highest for unrelated groups and lowest for patient groups. However, there were no statistically significant differences between Lamin A and Lamin C expression across all samples (FDR adjusted *p*-value ≥0.05); **b** RNA-seq validation of Lamin A (LMNA) and Lamin C (LMNC) transcript levels by quantitative PCR (qPCR). qPCR was performed on cDNA generated from unrelated, control and patient fibroblasts to measure Lamin A and Lamin C transcript levels (average QGE +/− standard error of the mean (SEM)). There were no statistically significant differences across all groups in transcript levels for Lamin A [F (2,6)= 0.90, *p* = 0.46] and for Lamin C [F (2,6)= 1.76, *p* = 0.25], validating trends observed in RNA-seq. **c** Western blot for Lamin A/C protein. Lamin A/C protein levels were measured by immunoblotting using antibodies specific against Lamin A/C and Beta Actin (upper panel). Full-length images are available in the supplementary information (Additional File 2: Figure S3). Quantification of Lamin A/C bands relative to Beta Actin (lower panel) showed no significant differences for average AU +/− SEM between groups [Lamin A: F (2,6)= 0.95, *p* = 0.44 and Lamin C: F (2,6)= 1.63, *p* = 0.27]. Statistical analysis was performed using One-way ANOVA followed by Tukey post hoc. AU = Absorbance Unit

**Fig. 3:**
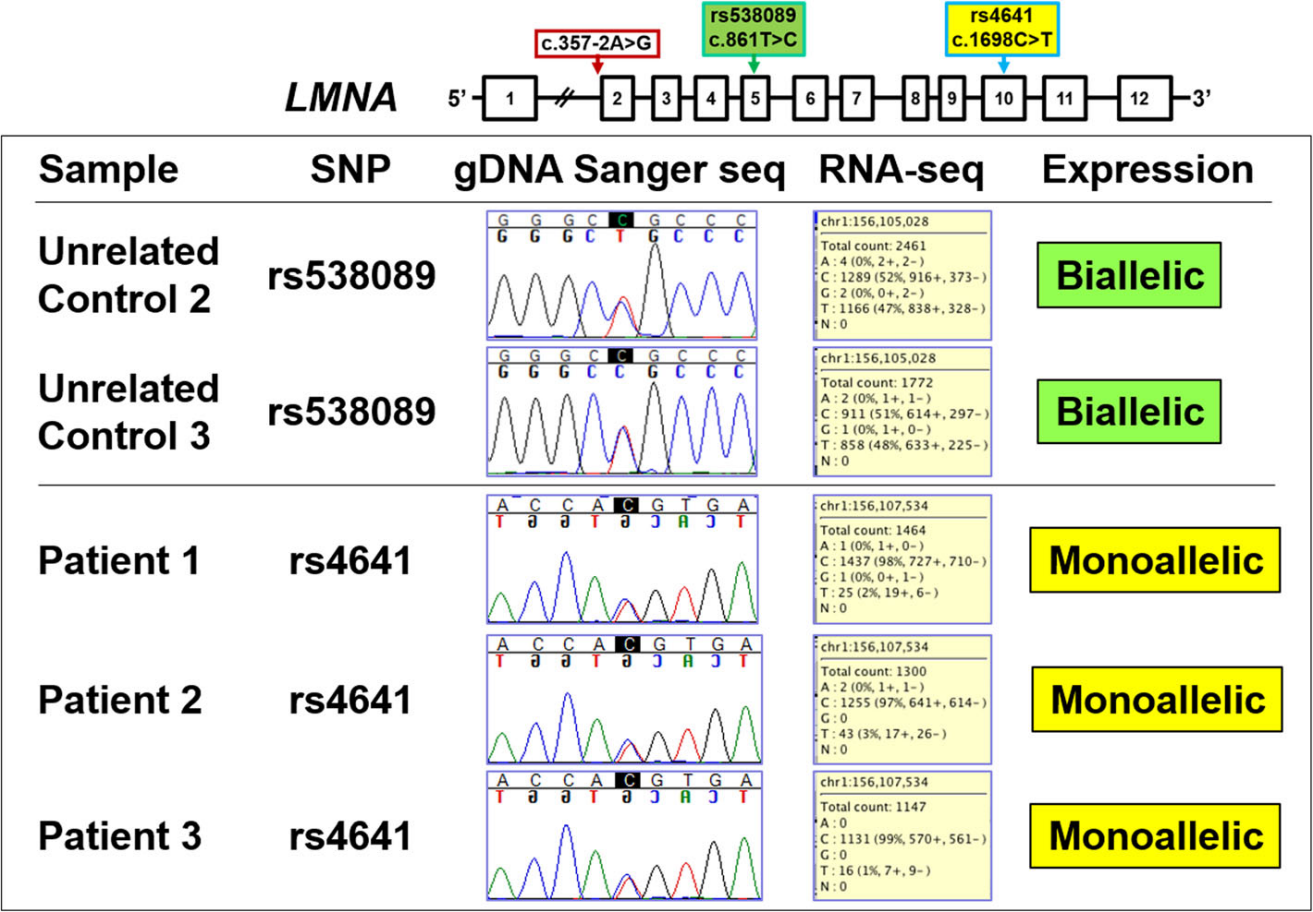
*LMNA* allelic expression. Top panel depicts location of the *LMNA* splice-site mutation and two expressed single nucleotide polymorphisms (SNP), rs538089 in Exon 5 and rs4641 in Exon 10. Bottom table shows Sanger sequencing chromatograms from genomic DNA (gDNA) and distribution of allele read counts from RNA-seq. For two Unrelated Controls at rs538089, gDNA was heterozygous (C/T) with equal RNA-seq reads of C (51–52%) and T (47–48%) consistent with biallelic expression. For three Patients at rs4641, gDNA was heterozygous (C/T) with RNA-seq reads predominately of C (97–99%) consistent with monoallelic expression

Because we hypothesized that the expression of the *LMNA* isoforms may be influenced by the *LMNA* mutation in the patient samples, we examined the expression of the *LMNA* isoforms individually (Fig. 2). As seen in the heatmap and RNA-seq data (Fig. 2a, Additional File 1: Table S3), although the patients have the *LMNA* mutation we did not see significant DE between the sample groups for the *LMNA* isoforms. Next, we investigated whether the predicted exon skipping and aberrant splicing occurred in patient samples by examining the aligned sequences across the *LMNA* transcript. Here, we observed sequence alignment that corresponds to the presence of exon 2 of *LMNA* transcript and similar read coverage across all of the *LMNA* exons for each patient sample (Additional File 2: Figure S1). Therefore, our data is consistent with the lack of exon skipping and aberrant splicing in mutant fibroblasts. We confirmed the RNA-seq data for the nuclear lamina associated genes using qPCR and found no statistically significant differences in Lamin A and Lamin C (Fig. 2b) as well as Lamin B1 and Lamin B2 expression between all sample groups (Additional File 2: Figure S2). Validation by Western Blot showed that the amount of Lamin A/C proteins did not vary significantly between the Patient and the two control groups (Fig. 2c). In mutant fibroblasts, we also used RNA-seq to confirm that *LMNA* expression was predominately from the wild type allele [35] (Fig. 3).

### ERK/MAPK signaling pathway identified by DE and pathway analysis

**Fig. 4:**
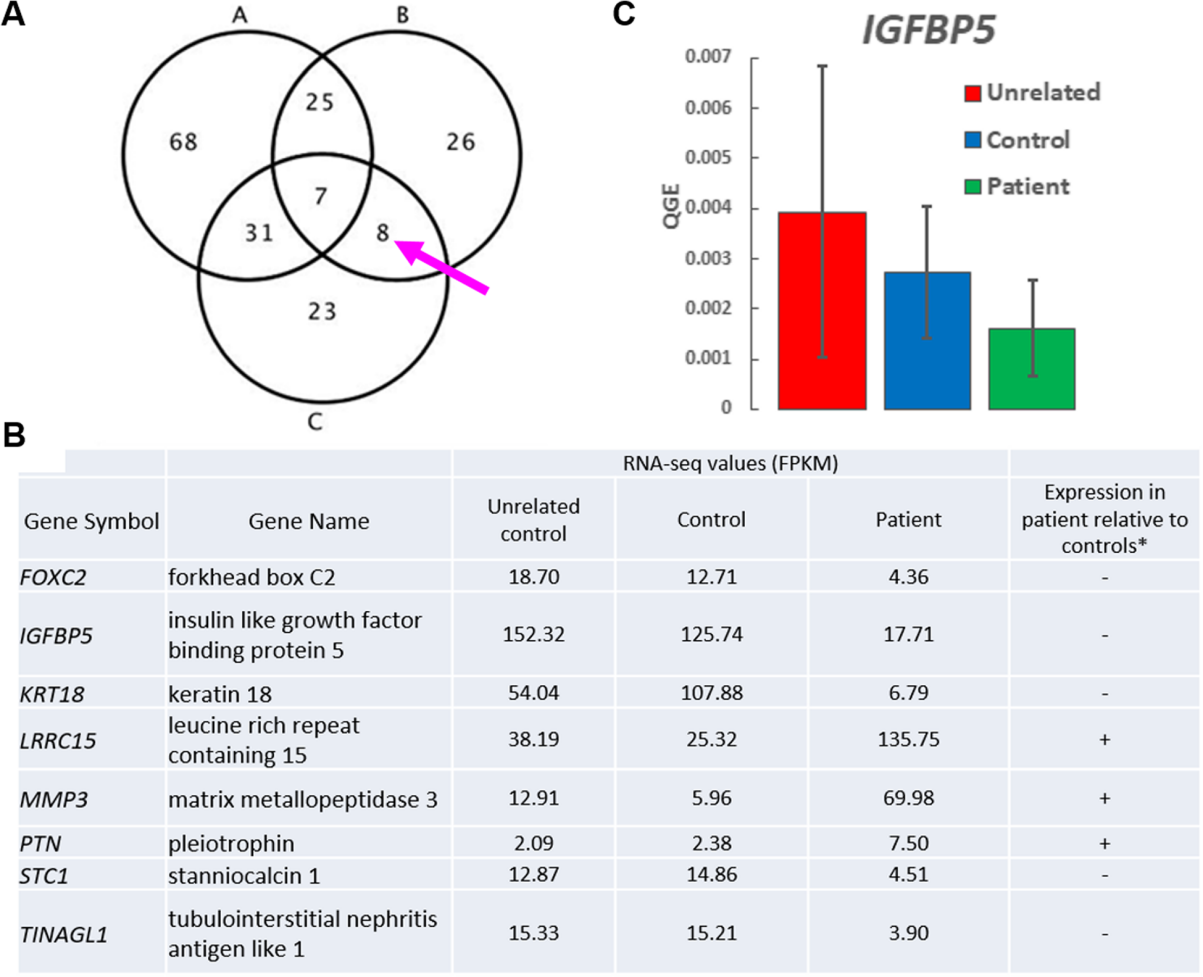
Eight differentially expressed genes are potentially involved in *LMNA*-related cardiomyopathy. **a** Differentially expressed genes that passed the filtering criteria were input into Ingenuity Pathway Analysis (IPA). Of these significantly differentially expressed genes for each group, there were eight genes in the intersection (arrow) between B and C, but not A, where A = Unrelated vs. Controls, B = Unrelated vs. Patients, C = Controls vs. Patients; **b** Gene symbols, names, and RNA-seq values for eight genes found in the intersection between groups B and C, but not A; *Expression in patient: -: decreased, +: increased. **c** RNA-seq validation of Insulin Growth Factor binding protein 5 (*IGFBP5*) transcript levels by quantitative PCR (qPCR). qPCR was performed on cDNA generated from unrelated, control and patient fibroblasts to measure *IGFBP5* transcript level. There was no statistically significant difference in IGFBP5 transcript levels (average QGE +/− SEM) between all groups [F (2,6)= 0.37, *p* = 0.71]. Statistical analysis was performed using One-way ANOVA followed by Tukey post hoc test

**Fig. 5:**
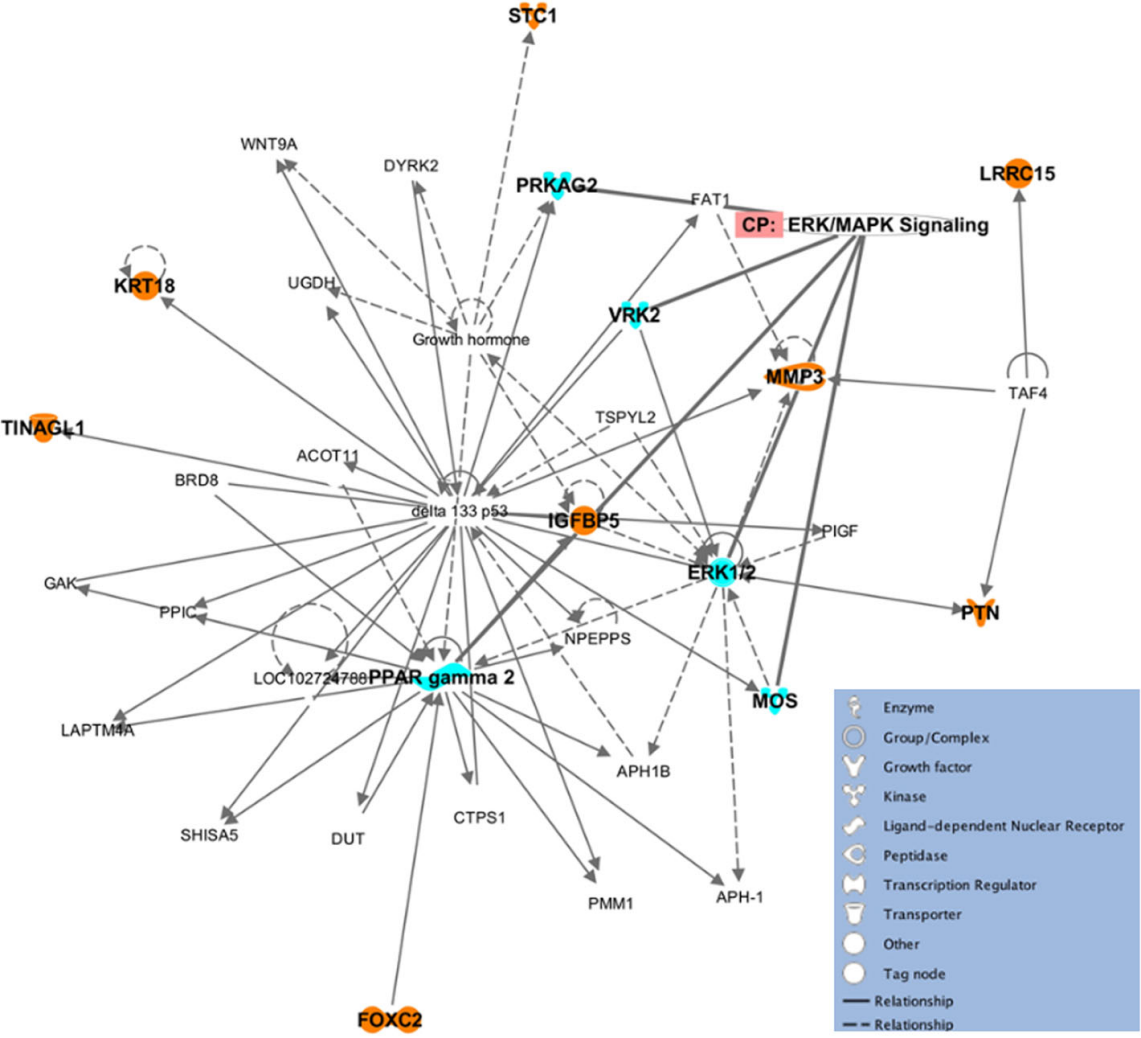
Ingenuity Pathway Analysis (IPA) of network of eight DE genes. The eight genes that were found by the intersection shown in Fig. 3 were input into IPA to generate a network diagram with the most plausible connections to each other, and to additional genes in the IPA Knowledge Base. The eight genes of interest are highlighted in orange. A maximum of thirty-five genes are shown in the network for readability. The ERK/MAPK pathways genes found in this network are highlighted in cyan

Next, we turned our attention to other genes that were most significantly upregulated or downregulated between Patients, Controls, and Unrelated Controls. We found eight DE genes that were similarly expressed between the three groups (Fig. 4a, b). Among these eight DE genes were genes necessary for proper fibroblast physiological function such as Matrix Metallopeptidase 3 (*MMP3*) [45] and for epithelial-to-mesenchymal transition such as Keratin 18 (*KRT18*) [46]. In addition, we found one DE gene that was involved in the insulin-like growth factor (IGF) signaling pathway: *IGFBP5*. This gene was of particular interest due the known effects of Lamin A/C on the IGF pathway in different forms of laminopathies [47]. Previous gene expression studies by microarray have reported *IGFBP5* mRNA level to be downregulated in *Lmna* −/− mouse embryonic fibroblasts, compared to *Lmna* +/+ mouse embryonic fibroblasts [48]. We also tested the DE gene expression in the RNA-seq data by performing qPCR on the *IGFBP5* gene (Fig. 4c). The qPCR result showed similar trend as the RNA-seq data with Patient *IGFBP5* gene expression decreased by greater than two-fold compared to Unrelated Controls. Finally, we used pathway analysis to demonstrate that these eight DE genes were connected in a gene network that included ERK/MAPK pathway genes (Fig. 5). This finding showed the connectivity of the DE genes in a signaling network and demonstrated the possibility of this pathway being affected in fibroblasts from *LMNA*-related cardiomyopathy patients.

## Discussion

### Compensatory role of IGFBP5 in maintaining ERK/MAPK pathway homeostasis in *LMNA* mutant fibroblasts

Here we report gene expression profiling using RNA-seq on fibroblasts from a family bearing a pathogenic *LMNA* splice-site mutation. At present, the molecular mechanisms regulating tissue-specific manifestations of laminopathies are still unidentified. However, multiple studies have shown that *LMNA* mutations cause nuclear envelope defects in mouse and human *LMNA* mutant fibroblasts [49–51] and dysregulation of molecular signaling such as ERK/MAPK and Wnt Beta-Catenin pathways in mutant cardiac tissues [9, 10]. In human cell lines, cultured cells from patients, and pluripotent stem cell disease models for *LMNA*-related diseases, upregulation of the ERK/MAPK pathway also is documented [52, 53]. Taken together, these studies suggested that laminopathies may arise from a combination of defects in the nuclear structure and the dysregulation of molecular signaling pathways in mutant cells.

Consistent with this, our gene expression profiling uncovered genes involved in signal transduction that are differentially expressed between *LMNA* mutant and normal human fibroblasts. Among eight significantly DE genes was the *IGFBP5* gene that encodes for an IGF binding protein that stabilizes IGF cytokines to promote or inhibit its signaling cascade and subsequent IGF function in cell culture [54]. Aside from its cytokine binding function, IGFBP5 protein overexpression was shown to suppress cell growth in human melanoma, osteosarcoma, and breast cancer cell lines emphasizing its function in regulating cell proliferation [55–58]. Overexpression of IGFBP5 also was shown to enhance osteogenic differentiation in mesenchymal stem cells [59]. These observations resulted in increased phosphorylation of ERK1/2 and increased activity of the MAPK pathway, and predictably IGFBP5 knock down by shRNA reduced phosphorylation of ERK1/2 and subsequently downregulated the MAPK pathway [58–60]. Subsequently, our pathway analysis showed that the eight DE genes, including *IGFBP5,* were connected through the ERK/MAPK pathway. Our RNA-seq data showed a significant reduction in *IGFBP5* gene expression in patient samples compared to control (7-fold reduction) and unrelated samples (9-fold reduction). This reduction may potentially impact the signaling pathways downstream to IGFBP5 binding. IGFBP5 protein translocates to the nucleus and interacts with Early growth response-1 transcription factor in human primary lung fibroblast to induce extracellular matrix production by activating the ERK/MAPK pathway, independent of the IGF pathway [61]. Since we did not detect any significant changes in the expression of canonical IGFBP5 binding partners, such as IGF1 and IGF2, or in IGF pathway activity, this suggests that the IGF pathway maintained its normal activity in patient fibroblasts. Taken together, these observations along with findings presented in published studies suggested that IGFBP5 exerts its effect through the ERK/MAPK pathway rather than the IGF pathway.

With these findings, we hypothesize that in unaffected tissues such as *LMNA* mutant fibroblasts, the significant reduction of IGFBP5 expression may contribute to maintaining homeostasis of the ERK/MAPK signaling pathway and its downstream effects, masking the *LMNA* mutation phenotype. The postulation is supported by the observations of normal baseline ERK1/2 activity in patient fibroblasts [53]. In contrast, there is aberrant upregulation of ERK/MAPK pathway in affected tissues such as *LMNA* mutant cardiomyocytes which leads to laminopathy phenotype [10, 62]. Together, these mechanisms may provide an explanation for the absence of any significant phenotype in fibroblasts [63].

Another DE gene on the list, Forkhead Box C2 (*FOXC2*), whose overexpression induces hyperphosphorylation of ERK1/2 in cancer cells and is associated with aberrant cell proliferation [64], also has decreased mRNA expression in the affected individuals. *FOXC2* mRNA reduction by shRNA silencing has been shown to downregulate ERK/MAPK activity [64]. The downregulation of *IGFBP5* and *FOXC2* gene expression may contribute to the compensatory mechanism that maintains the normal activity of ERK/MAPK pathway and absence of observable phenotypes in fibroblasts. While the current study was performed on an unaffected tissue, we were able to gain insight on the possible signaling pathways and molecular mechanisms involved in the tissue specificity of laminopathies and *LMNA*-related cardiomyopathy.

### Normal Lamin A/C protein levels by monoallelic expression and no compensation by B-type lamins in mutant fibroblasts

Previously, we showed that there was monoallelic expression of *LMNA* gene in our patient fibroblasts, whereby only the wild type allele was expressed, and we predicted that the mutant allele expression was degraded by non-sense mediated mRNA decay [35]. In support of this, our RNA-seq results also showed *LMNA* expression was predominately from the wild type allele (97–99%) with no aberrantly spliced products from the mutant allele in patient fibroblasts.

We also found that there were no significant changes in *LMNA* transcript levels, ratio of Lamin A to Lamin C transcripts, or expression of other NL-genes, *LMNB1* and *LMNB2* between the Patient group and two control groups. These results are in line with our previous structural studies that found no significant difference in nuclear abnormalities and suggests that there were no compensatory mechanisms at the transcription level for the other proteins that construct the nuclear lamina [63].

Our results are in contrast to previous studies in patient fibroblasts that showed decreased Lamin A/C expression and altered ratio of Lamin A and Lamin C expression [65, 66]. Real-time PCR studies on fibroblasts from patients with a heterozygous *LMNA* mutation at the splice site of intron 5 showed reduced *LMNA* expression with the mutant *LMNA* allele expressed at lower level compared to the normal allele [65]. In a different study, Lamin A protein expression was more diminished compared to Lamin C protein in fibroblasts from patients with Lamin-associated cardiomyopathies [66].

Numerous studies have reported that differential gene expression does not correlate directly with changes in protein expression [67–70]. Muchir et al. has previously reported that fibroblasts from EDMD, DCM and LGMD1B patients bearing heterozygous *LMNA* mutations have similar Lamin A/C protein levels compared to control fibroblasts [51]. Additional Lamin A/C protein studies in patient fibroblasts have also shown that heterozygous *LMNA* mutations do not result in significantly observable phenotypes such as nuclear abnormalities or reduced protein level [52, 71–73].

Here, we performed validation studies for Lamin A/C protein expression using Western Blot to resolve these discrepancies. We found that there was no significant differences in Lamin A/C protein expression in mutant fibroblasts. This contrasts the decreased or absent levels of Lamin A/C protein detected previously in patient cardiac tissues [74, 75]. Thus, our results suggest that there is upregulated expression of wild type *LMNA* allele in response to the degradation of the mutant allele or that expression of only one copy of the wild type allele is sufficient for normal levels of Lamin A/C protein to maintain normal physiological function in unaffected cell types. Other potential molecular mechanisms, such as epigenomic alterations specific to patient cardiac tissue [34, 76], tissue specific expression of Lamin proteins [17, 77], or differences in nuclear membrane proteome between tissues [78], may contribute to limit Lamin A/C haploinsufficiency to diseased cardiac tissues. Further studies comparing affected and unaffected tissues derived from the same patient or from patient-specific disease models may provide additional insight to the molecular mechanism of this mutation.

### Gene expression variabilities exist in samples within and between groups

We obtained in total nine dermal fibroblast samples from affected, related unaffected, and unrelated individuals to perform RNA-seq. Though we age-matched and gender-matched the patients to controls to the best of our abilities, we found significant variability between samples within groups. While age is thought to be a factor that can influence gene expression [79], recent study on cultured fibroblasts in-vitro harvested from young, middle aged and older individuals revealed no significant difference in gene expression based on age groups [80]. The similarity between the published study and ours led us to conclude that short term in-vitro culture conditions are not sufficient to induce gene expression differences that are influenced by age.

Epigenomic modifications play a role in modulating gene expression [81–83]. We postulate that expression differences may arise from different degrees of epigenetic modifications accumulated or inherited by the individuals studied here throughout their lifetime. Our qualitative observations indicated that variabilities intrinsic to the samples must be taken into consideration when performing gene expression analysis. At present, we believe the expression differences we observed here are due to the small sample size available for our studies. These differences must be taken into account for future gene expression profiling studies by increasing our sample sizes and technical replicates per group.

## Conclusions

Using deep RNA-seq and pathway analysis, we identified eight differentially expressed genes in fibroblasts from an affected family with a novel *LMNA* splice-site mutation and heart disease. Among these eight genes, we identified *IGFBP5* as one of the candidate genes affected by *LMNA* mutation and propose a possible molecular mechanism that explains the lack of significant molecular and structural phenotype in unaffected tissues. The identification of the ERK/MAPK pathway connecting the candidate genes together supports published studies that implicate this pathway in the development of *LMNA*-related cardiomyopathy. Our results for normal Lamin A/C protein and *LMNA* gene expression from only the wild type allele in mutant fibroblasts also suggest that cardiac tissues are more sensitive to decreases in Lamin A/C protein.

We are aware of the limitations of our studies as presented here. The variation of gene expression within and between sample groups introduced confounding factors to our analysis and our use of an unaffected tissue (fibroblasts) instead of affected tissue (cardiomyocytes) result in a partial explanation of the molecular mechanisms of this particular *LMNA* splice-site mutation. Thus, our results indicate the necessity to develop a model system to study this disease in vitro. To that end, we will generate patient-specific induced pluripotent stem cells lines that will be differentiated to cardiomyocytes for molecular studies to confirm the proposed tissue-specific mechanism of disease.

## Supplementary information

**Additional file 1: Table S1**. Sequencing statistics. **Table S2**. qPCR primer sequences. **Table S3**. RNA-seq expression values for *LMNA*, *LMNC*, and *IGFBP5.*

**Additional file 2: Figure S1**. IGV screenshots showing expression of *LMNA* transcripts at exons 1, 2 and 3. **Figure S2**. Lamin B1 and Lamin B2 heatmap and qPCR validation. **Figure S3**. Original Lamin A/C Western Blot.

## Data Availability

The RNA-seq dataset supporting the conclusions of this article is available from the NCBI Gene Expression Omnibus (GEO), Accession: GSE125990, and accessible via the following link: [https://www.ncbi.nlm.nih.gov/geo/query/acc.cgi?acc=GSE125990]. In addition, the dataset for Human reference genome (GRCh37/hg19) is available from the National Center for Biotechnology Information via the following link: [https://www.ncbi.nlm.nih.gov/assembly/GCF_000001405.13/].

## Abbreviations

NL: Nuclear lamina
DCM: Dilated cardiomyopathy
EDMD: Emery-Dreyfus Muscular Dystrophy
LGMD1B: Limb Girdle Muscular Dystrophy type 1B
RNA-seq: RNA sequencing
P: Patient
C: Control
U: Unrelated Control
gDNA: Genomic DNA
DE: Differential expression
FPKM: Fragments Per Kilobase of transcript per Million mapped reads
FDR: False discovery rate
IGV: Integrative Genomics Viewer
IPA: Ingenuity Pathway Analysis
qPCR: Quantitative PCR
QGE: Quantitative gene expression
IGF: Insulin-like growth factor
SEM: Standard error of the mean
