# Supplementary material for: Gene expression profiling of fibroblasts in a family with LMNA-related cardiomyopathy reveals molecular pathways implicated in disease pathogenesis
Source: BMC Med Genet. 2020 Jul 22;21:152. doi: 10.1186/s12881-020-01088-w (PMC7374820; doi:10.1186/s12881-020-01088-w)
Supplement: Supplementary file 1 — Additional file 1: Table S1. Sequencing statistics. Table S2. qPCR primer sequences. Table S3. RNA-seq expression values for LMNA, LMNC, and IGFBP5. [file 12881_2020_1088_MOESM1_ESM.pdf]

Table S1: Sequencing statistics

Group	SampleID	Total Number of Reads (left+right)	Overall read mapping rate	Concordant Pair Alignment Rate
Unrelated controls	RNA1_U1	116,415,950	88.6%	83.6%
	RNA2_U2	119,163,654	90.8%	86.2%
	RNA3_U3	127,339,996	92.4%	88.1%
Controls	RNA4_C1	119,696,150	88.9%	84.0%
	RNA5_C2	123,096,068	89.4%	84.7%
	RNA6_C3	112,137,798	89.1%	84.4%
Patients	RNA7_P1	159,013,354	88.8%	84.0%
	RNA8_P2	142,759,722	91.9%	87.3%
	RNA9_P3	140,901,146	91.4%	87.1%

Table S2: qPCR primer sequences

**Gene name (Symbol)**	**Primer sequence (5'→ 3')**
Lamin A (*LMNA*)	CTC CAC ATC TGC CTT AAA AC<br>GCT AGC CTC TAT AAA AGC AC
Lamin C (*LMNC*)	AGA ACA TCT ACA GTG AGG AG<br>CAG AAT AAG TCT TCT CCA GC
Lamin B1 (*LMNB1*)	GCG TGC GTG TCT ATG CTA AG<br>TCA TGC GGC TTT CCA TCA GT
Lamin B2 (*LMNB2*)	TTT TCC ACC AAC AGG GGG AC<br>ACG TTC TGG CAG TTC GCT TA
Insulin Growth Factor binding protein 5 (*IGFBP5*)	GCC CTC CAC CTC TCT CTA CA<br>TCA CGG GAG TCT CTC TCG AT
Housekeeping gene: Beta actin (*ACTB*)	GAC GAC ATG GAG AAA ATC TG<br>ATG ATC TGG GTC ATC TTC TC

Table S3: RNA-seq expression values for *LMNA*, *LMNC*, and *IGFBP5*

**Sample**	**LMNA_NM_170707**	**LMNC_NM_005572**	***LMNA* to *LMNC* ratio**	**IGFBP5_NM_000599**
All Unrelated Controls	124.946	300.189	0.416	152.317
U1	122.652	322.642	0.380	26.8803
U2	136.26	343.83	0.396	57.5047
U3	115.927	234.096	0.495	372.565
All Controls	116.521	280.476	0.415	125.739
C1	127.337	317.053	0.402	65.8477
C2	112.905	273.745	0.412	127.077
C3	109.32	250.63	0.436	184.293
All Patients	67.6258	172.028	0.393	17.7081
P1	69.795	196.989	0.354	9.63751
P2	72.2225	171.963	0.420	5.8749
P3	60.8598	147.132	0.414	37.6119
