# Supplementary material for: Gene expression profiling of fibroblasts in a family with LMNA-related cardiomyopathy reveals molecular pathways implicated in disease pathogenesis
Source: BMC Med Genet. 2020 Jul 22;21:152. doi: 10.1186/s12881-020-01088-w (PMC7374820; doi:10.1186/s12881-020-01088-w)
Supplement: Supplementary file 2 — Additional file 2: Figure S1. IGV screenshots showing expression of LMNA transcripts at exons 1, 2 and 3. Figure S2. Lamin B1 and Lamin B2 heatmap and qPCR validation. Figure S3. Original Lamin A/C Western Blot. [file 12881_2020_1088_MOESM2_ESM.pdf]

Figure S1

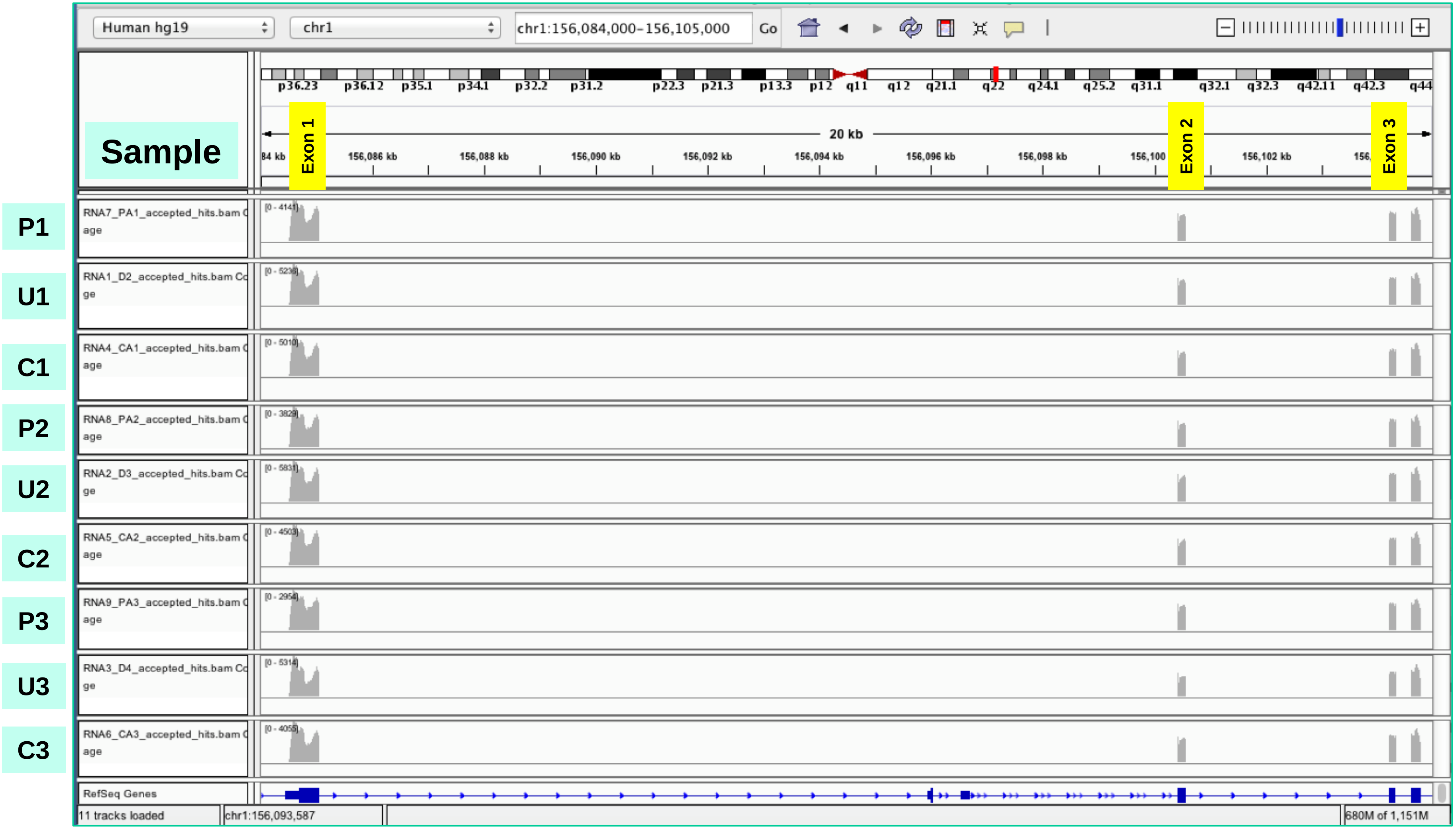

Figure S1. Integrative Genomics Viewer (IGV) screenshots showing expression of *LMNA* transcripts at Exons 1, 2 and 3 for all samples. There was a peak at each exon, indicating similar read coverage across all *LMNA* exons for all samples.

Figure S2

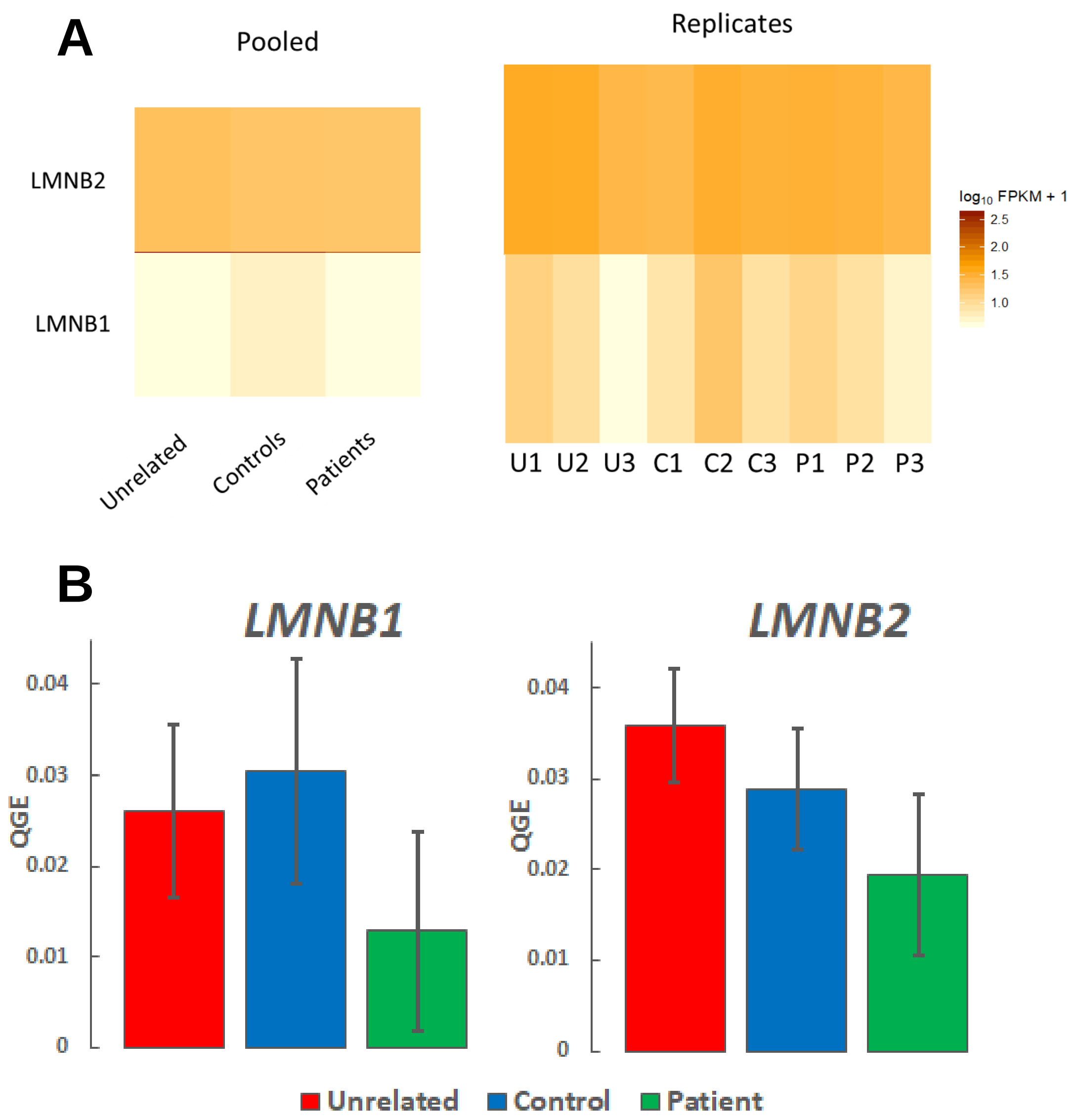

Figure S2. Lamin B1 and Lamin B2 heatmap and qPCR validation**. a** Heatmap showing Lamin B1 (*LMNB1*) and Lamin B2 (*LMNB2*) expression across all samples as pooled (left panel) and with replicates shown (right panel). There was no significant difference in the expression of *LMNB1* and *LMNB2* across all samples (FDR-adjusted p-value ≥ 0.05). **b** RNA-seq validation of Lamin B1 and Lamin B2 transcript levels by quantitative PCR (qPCR). qPCR was performed on cDNA generated from unrelated control, control and patient fibroblasts to measure Lamin B1 and Lamin B2 transcript levels. There were no statistically significant differences in transcript levels (average QGE +/- standard error of the mean (SEM)) between groups for Lamin B1 [F(2,6)= 0.70, p=0.53] and for Lamin B2 [F(2,6)= 1.29, p=0.34]. Statistical analysis was performed using One-way ANOVA followed by Tukey post hoc test.

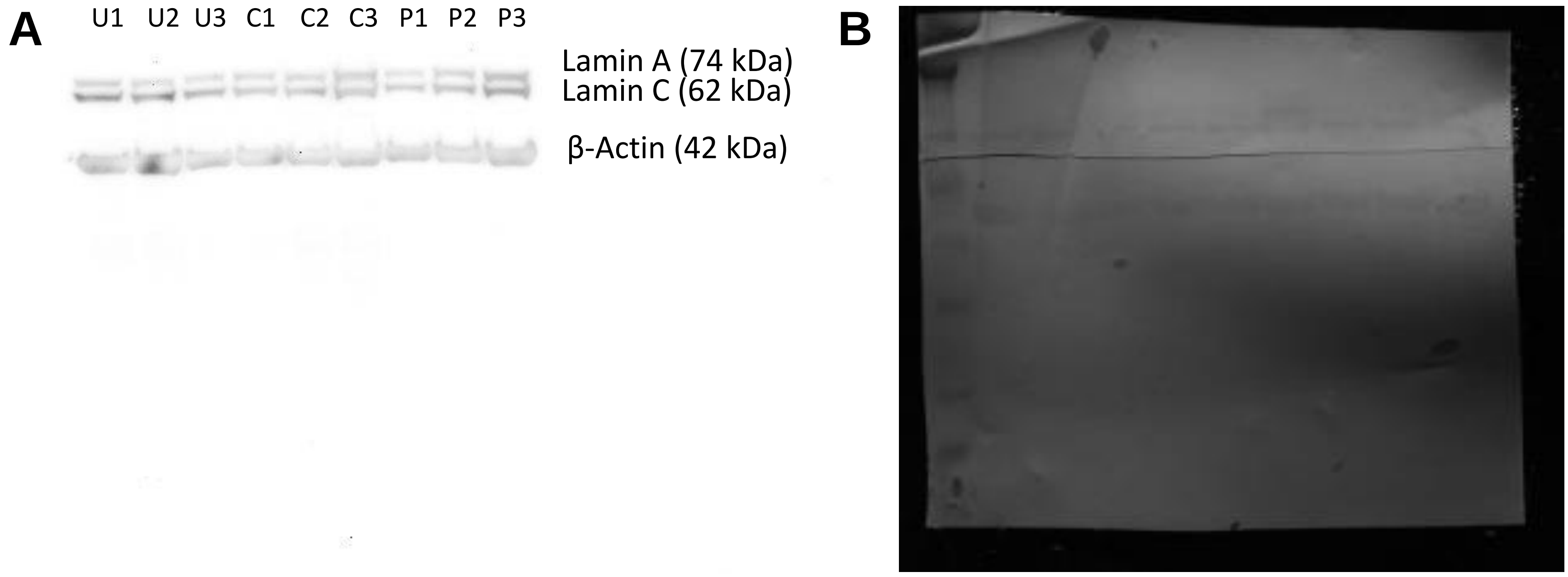

Figure S3. Original Lamin A/C Western Blot. **a** Fibroblasts from three unrelated (U1-U3), three control (C1-C3) and three patient (P1-P3) samples were seeded and grown to confluency, then total protein lysates were extracted for Western blot. Immunoblotting were conducted using N-terminus Lamin A/C antibody and β-Actin as loading control. **b** Membrane image under white light illumination.
